# A Critical Review of Electrochemical Glucose Sensing: Evolution of Biosensor Platforms Based on Advanced Nanosystems

**DOI:** 10.3390/s20216013

**Published:** 2020-10-23

**Authors:** Vuslat B. Juska, Martyn E. Pemble

**Affiliations:** 1 Tyndall National Institute, University College Cork, T12 R5CP Cork, Ireland; martyn.pemble@tyndall.ie; 2 School of Chemistry, University College Cork, T12 YN60 Cork, Ireland

**Keywords:** glucose sensors, nanomaterials, electrochemical biosensors

## Abstract

The research field of glucose biosensing has shown remarkable growth and development since the first reported enzyme electrode in 1962. Extensive research on various immobilization methods and the improvement of electron transfer efficiency between the enzyme and the electrode have led to the development of various sensing platforms that have been constantly evolving with the invention of advanced nanostructures and their nano-composites. Examples of such nanomaterials or composites include gold nanoparticles, carbon nanotubes, carbon/graphene quantum dots and chitosan hydrogel composites, all of which have been exploited due to their contributions as components of a biosensor either for improving the immobilization process or for their electrocatalytic activity towards glucose. This review aims to summarize the evolution of the biosensing aspect of these glucose sensors in terms of the various generations and recent trends based on the use of applied nanostructures for glucose detection in the presence and absence of the enzyme. We describe the history of these biosensors based on commercialized systems, improvements in the understanding of the surface science for enhanced electron transfer, the various sensing platforms developed in the presence of the nanomaterials and their performances.

## 1. A Brief History of Glucose Biosensing

**Figure 1:**
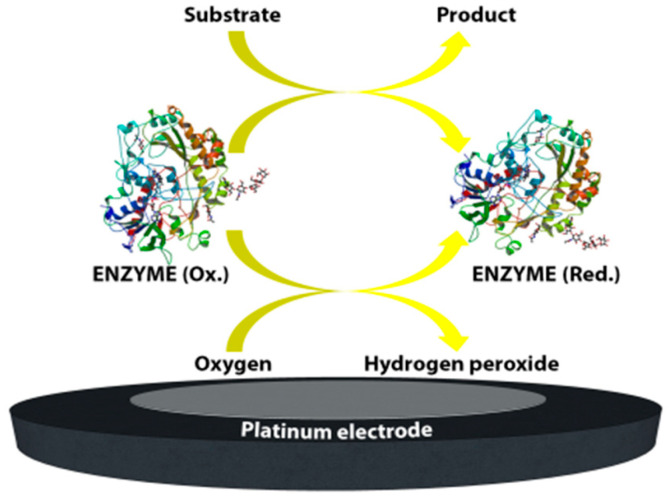
A schematic representation of first-generation glucose.

The first enzyme-based electrode for glucose detection was reported by Clark and Lyons in 1962 [1]. This device was based on glucose oxidase entrapped within a semipermeable dialysis membrane which was constructed on an oxygen electrode. Following this, Clark’s patent in 1970 demonstrated the use of enzymes to convert electroinactive substrates to electroactive substances (Figure 1). This system relied on two different electrode systems. The first electrode system, which consisted of at least one enzyme in a capillary thin layer between the electrode and the membrane, was responsible for the conversion of the substrate to electroactive material in the presence of interfering species. The second electrode system was sensitive to the interfering species available in the sample. By subtracting the measured current from the second electrode system from the measured current from the first electrode system, Clark’s patented device was successful to monitor the glucose. 

**Figure 2:**
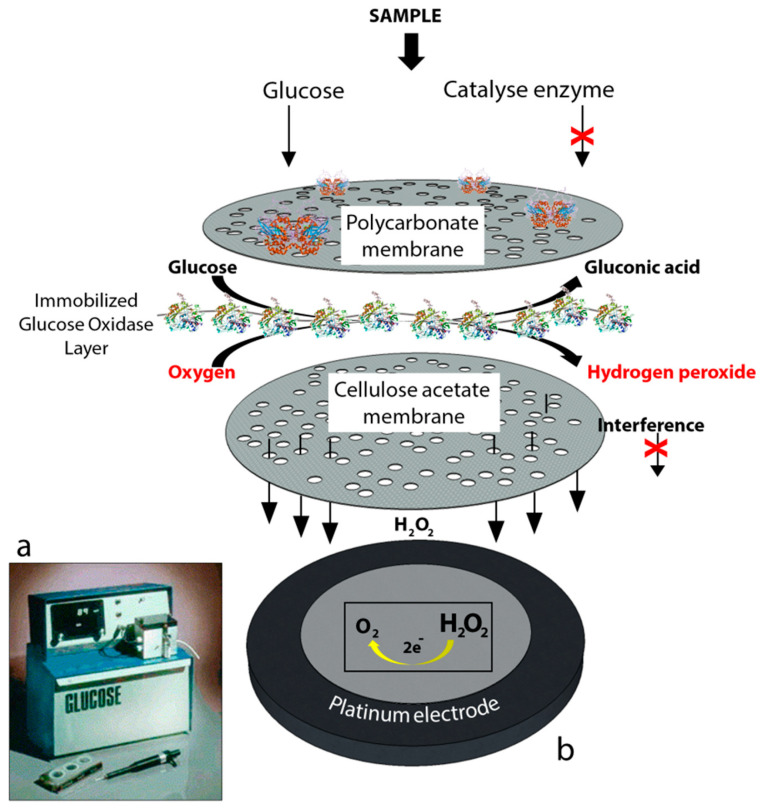
(**a**) YSI 23A glucose biosensor [2] and (**b**) sensor probe with immobilized enzyme membrane for the Yellow Springs Instruments (redrawn from Paul D’Orazio, Biosensors in clinical chemistry [3]).

First generation biosensors: Clark’s technology was then transferred to the Yellow Springs Instrument Company and became a commercial product in 1975 with the successful launch of the first glucose analyzer based on the amperometric detection of hydrogen peroxide from the samples of whole blood, the so-called model 23A YSI analyzer, Figure 2.

The YSI 23A biosensor was also based on Clark’s electrode scheme. However, it relied on hydrogen peroxide oxidation for glucose monitoring since hydrogen peroxide is also produced by the enzymatic reaction at a concentration which is proportional to the glucose concentration. Briefly, for the construction of the biosensor, glucose oxidase was immobilized between two membrane layers. The first layer was a polycarbonate membrane which was used to permit only glucose molecules to move towards the enzyme layer by blocking the many other larger substances including enzymes and proteins available in whole blood; thus, decreasing the interference effect of the species. Therefore, only glucose reached the enzyme layer where it was oxidized. The product hydrogen peroxide from this reaction passed through a cellulose acetate membrane which also acted as a barrier for the larger molecules. Finally, hydrogen peroxide was amperometrically detected at the platinum electrode surface. The associated reactions are shown below:

(1) $$\mathrm{Reaction}\;1:\;\mathrm{Glucose}\;+\;O_{2}\;\overset{Glucose\;oxidase}{\to }\;\mathrm{Gluconic}\;\mathrm{acid}\;+\;H_{2}O_{2}$$

(2) $$\mathrm{Reaction}\;2:\;H_{2}O_{2}\;\overset{+0.7\;V\;vs.\;Ag/AgCl}{\to }\;O_{2}\;+\;2H^{+}\;+\;2e^{-}$$

The first-generation biosensor of the “23A sensor probe” was relatively expensive due to the presence of a platinum electrode and the applied high voltage made the system prone to the effects of interfering species in the absence of the membranes [2].

Consideration of such issues in relation to the first commercial biosensor led to fierce competition in the market which in turn helped to establish the highly interdisciplinary research area of biosensors (and in particular electrochemical biosensors), which aims to improve the characteristics and performance of the biosensors in general and further miniaturize the overall systems, making them suitable for cheap, mass production.

**Figure 3:**
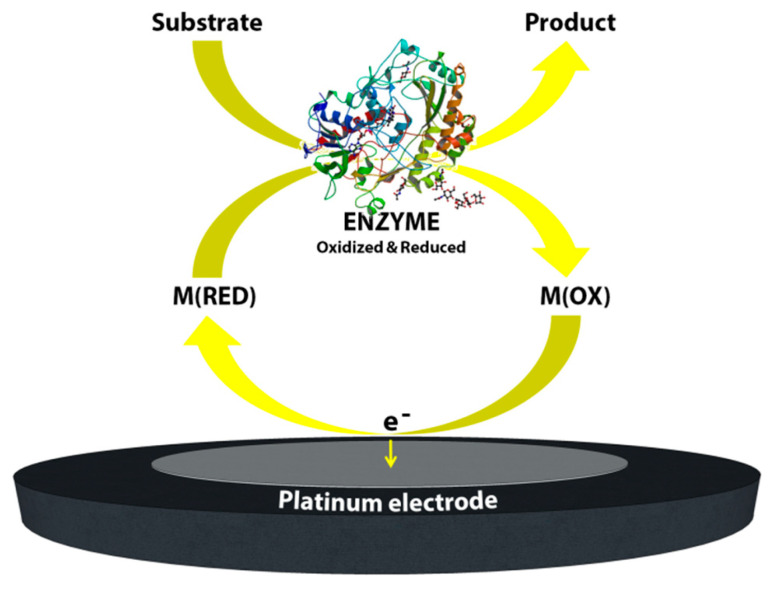
Schematic image of the mediated biosensor working principle, second generation biosensors.

Second generation biosensors: With this latter consideration in mind, the maturation of the fields of surface chemistry, screen-printing technologies and semiconductor integration technologies combined with the use of synthetic electron acceptors has resulted in major advances in the development of commercial electrochemical glucose biosensors. Synthetic electron acceptors in the form of redox couples or mediators are able to shuttle electrons between the redox center of the enzyme and the surface of the electrode. Thus, many inorganic redox couples and organic dyes have been successfully deployed in order to shuttle electrons for the reaction of glucose catalysis by glucose oxidase. Furthermore, it was shown that this method was efficient at lower applied potentials and thus provided decreased interference effects while also being insensitive to the dissolved oxygen concentration [2]. For these reasons in 1984 the first mediated amperometric biosensor toward glucose was reported [4]. Meanwhile, screen printing technologies were being adapted for the production of disposable, small or miniaturized, robust and cheap electrodes for amperometric biosensors. These two innovative research initiatives gave rise to the first very successful home-use blood glucose biosensor based on mediators and screen-printed electrodes. In 1987, these biosensors were launched under the brand name of ExacTech by the MediSense Company (original name Genetics International) which was a company founded between the universities of Cranfield and Oxford. Figure 3 shows a schematic of this first home-use glucose biosensor and illustrates its working principle. Arguably the application of mediators and screen-printed electrodes was a great step forward for the development of amperometric biosensors and commercial products for home use.

The “wiring” or “modification” of enzymes; towards third generation glucose biosensors: Even with the enormous success of the commercial, mediated glucose biosensors, the nature of the biochemical structure of the glucose oxidase enzyme, the relative solubility and toxicity of the mediators and the overall poor stability of these mediated systems towards extended continuous operation led researchers to concentrate on sensors based on direct electron transfer between the enzyme redox center and the electrode. The Flavin redox center of the enzyme (co-factor), which is deeply buried in an electrically insulated thick protein shell, is incapable of achieving an electrical connection with the electrode surface [5,6]. Thus, minimization of the electron-transfer distance is vital in order to ensure the performance of the sensor. Heller’s group reported one of the first smart routes to establishing this communication between the glucose oxidase active sites and the electrode using a long, flexible poly(4-vinlypyridine) (PVP) or poly(vinylimidazole) polymer backbone which had a dense array of linked osmium-complex electron relays [7]. In this way, the redox polymer penetrates and binds the enzyme and forms a three-dimensional network. This immobilization process—wiring the enzyme to the surface—significantly decreases the distance between the both redox centers of both the polymer matrix and the enzyme. Due to the permeable nature of the applied polymer, glucose and the product of the reaction are easily transferred between the matrix and the electrode. Another attractive route to facilitate electron transfer between the glucose oxidase redox center and the electrode surface is the chemical modification of the enzyme itself. In 1987, Degani and Heller [8] demonstrated the covalent attachment of ferrocenecarboxylic acid to the glucose oxidase via carbodiimide chemistry. When a sufficient amount of covalent attachment of ferrocenecarboxylic acid molecules to the enzyme was achieved, an enhanced electrical communication was obtained between the redox center of the enzyme and the electrodes (gold, platinum and carbon electrodes). In 1995, Willner’s group reported a highly elegant approach to improve the electrical contact by treating the glucose oxidase with electron relays [9]. In this study, the redox center of the glucose oxidase—flavin adenine dinucleotide (FAD)—was removed and modified with ferrocene. This was followed the reconstruction of the apo-enzyme with the ferrocene-modified FAD.

## 2. Enzymatic and Non-Enzymatic Electrochemical Biosensors

The various initiatives noted in the previous section have had a considerable influence on the development of the third-generation biosensors which are based on direct electron transfer in the absence of any kind of redox mediator. Such biosensors are able to operate at low applied potentials; thereby greatly reducing interference effects. However, as mentioned earlier, part of the challenge with these systems arises from the molecular structure of the enzyme itself. Many attempts have been made to try to create a successful matrix for the transfer of electrons between the FAD and the electrode by integration of conductive polymers and emerging nanomaterials such as carbon-based nanomaterials (carbon nanotubes [10,11,12,13,14], graphene [15,16,17], carbon/graphene quantum dots [18,19,20], etc.), metal nanoparticles [21,22,23,24,25] (gold, silver, copper, etc.), dendrimers [26,27], and many more.

### 2.1. Nanomaterial-Based Electrochemical Enzymatic Glucose Biosensors

**Figure 4:**
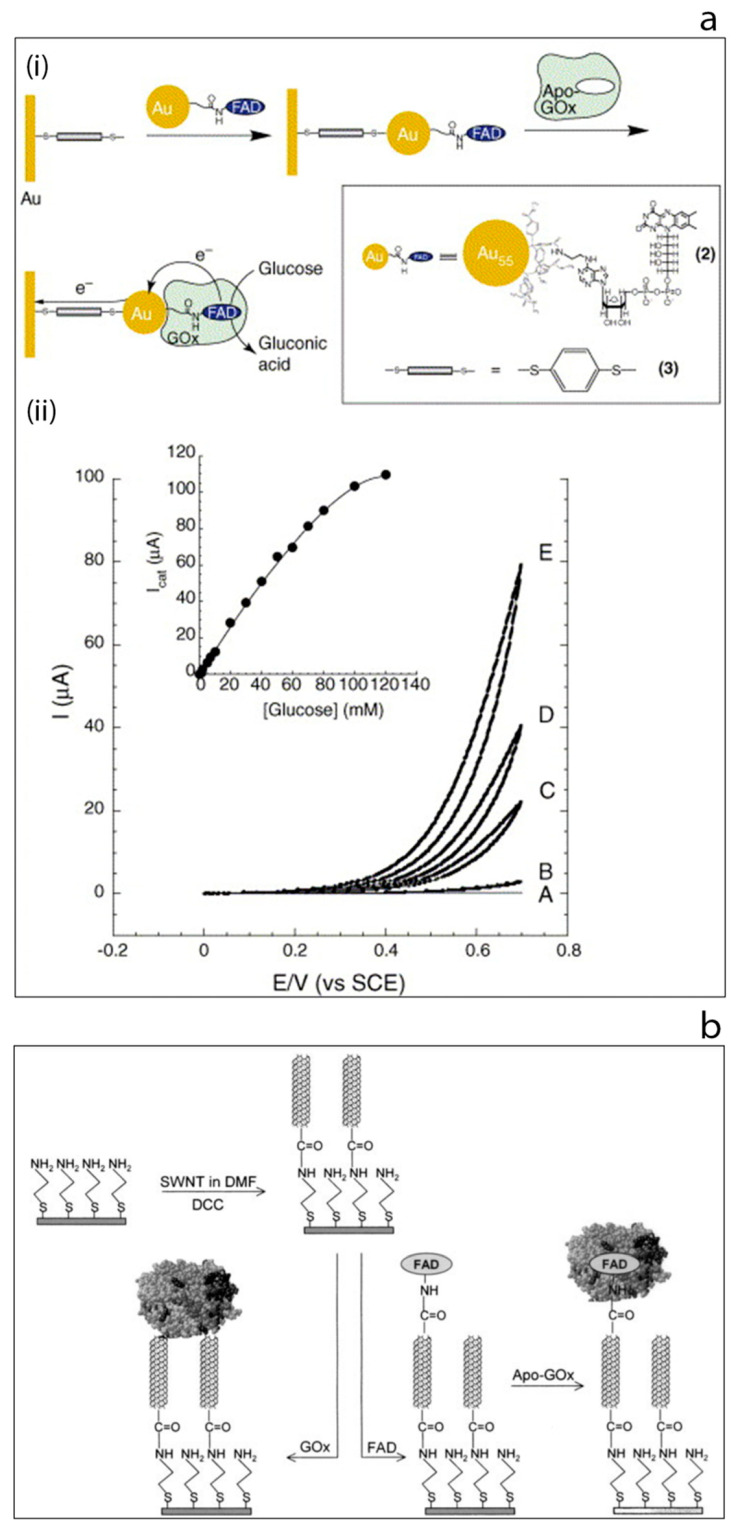
(**a**) Electrical wiring of glucose oxidase: (i) electrical contacting of glucose oxidase by reconstitution of apo-GOx on FAD-functionalized gold nanoparticles linked to an electrode surface by dithiol bridges, (ii) cyclic voltammograms obtained by the developed modified electrode in the presence of different glucose concentrations [37]. (**b**) Schematic image of the surface modification of gold electrode based on self-assembled monolayer with aligned single walled carbon nanotubes and their subsequent modification to allow direct electron transfer to glucose oxidase [43].

The enormous progress in the field of nanotechnology that has taken place over the past decade or so has been transferred to biosensors and bioelectronics in order to take advantage of some of the highly desirable features of nanomaterials. For example, in the context of biosensors, several methods have been developed for the synthesis of nanomaterials having with different shapes and dimensions such as spherical particles [19,20], rods [28,29], cubes [30], etc. In particular, carbon-based nanomaterials such as carbon nanotubes and graphene have given rise to major advances in the field [31,32,33,34,35,36]. The structural, electrical, chemical and mechanical properties of such nanostructures have made them an essential component of many detection technologies. Due to the similar size and dimensions of nanomaterials such as gold nanoparticles and carbon nanotubes to the redox enzymes, such nano structures might be used to establish a bridge between the electrode and the redox center as electrical connectors which might then enable improved electron transfer, Figure 4a [37,38,39,40]. An excellent example of this type of approach may be found by considering the work of the Willner group since they consecutively reported two interesting approaches for nanowiring of redox enzymes which have been accepted as seminal approaches by researchers in the field of biosensors and bioelectronics [41,42]. The first study which was published in 2003, used 1.4 nm diameter gold nanoparticles (carboxylic acid functionalized) to modify the electrode surface [41]. Then, a cofactor, aminoethyl-modified FAD, was immobilized onto the gold nanoparticles followed by the reconstitution of the apo-enzyme on the cofactor-functionalized gold nanoparticles. This study showed the ability of gold nanoparticles to act as relay units facilitating electron transport from the FAD to the electrode surface, thus activating the catalytic function of the enzyme [41]. Subsequently, in 2004 the same group published a second report which was based on the use of carbon nanotubes as molecular wires (‘nanoconnectors’) [42]. In this study, the aligned, reconstituted glucose oxidase on the edge of a single walled carbon nanotube was successfully linked to the surface thus ‘plugging’ the electrode into the enzyme. Electrons could then be transferred along the length of the carbon nanotubes. Such an enhanced direct connection between an enzyme and an electrode via aligned carbon nanotubes arrays was also reported by Gooding’s group in 2005, Figure 4b [43]. These pioneering studies from the Willner and Gooding groups encouraged researchers to further consider using nanostructures as the components of biosensors [41,42,43]. As a consequence, growth of research in the field of glucose biosensors has been phenomenal. It is noteworthy that the vast majority of this work in recent times is based on what might be termed ‘emerging’ nanomaterials.

#### 2.1.1. Gold Nanostructures and Their Use in Hybrid Glucose Biosensors

Gold nanostructures alone or in combination with other nanomaterials have been extensively studied in order to develop excellent matrixes for biomolecules and in particular those used for glucose oxidase immobilization [44,45]. For instance, nanoporous gold (NPG) has attracted significant interest in the field of electrochemical biosensors due to its large surface area and porosity.

In 2015, Wu et al. [46] reported a glucose biosensor based on a porous gold/enzyme combination. Briefly, in this work NPG was prepared by de-alloying 12-carat white gold leaves in concentrated HNO_3_. The resulting freshly-made NPG was immediately transferred onto a clean glassy carbon electrode surface and kept under vacuum. The NPG surface was later immersed into glucose oxidase solution for enzyme immobilization. The resulting GOx/NPG/GCE bio-electrode showed a sensitivity of 12.1 µA mM^−1^ cm^−2^ and a detection limit of 1.02 µM towards glucose detection. The success of this biosensor was attributed to the three-dimensional structure of the porous-gold matrix, which provided a good interface between the active sites of the enzyme and the electrode.

Rivas’s group demonstrated the use of globular gold nanoparticles as components of a nanohybrid structure by applying a series of chemical modifications to establish a highly efficient immobilization matrix for glucose oxidase [47]. For this purpose, gold nanoparticles were functionalized with 3-mercaptophenyl boronic acid (AuNPs-B(OH)_2_). A clean glassy carbon electrode was drop-casted with bamboo-like multi-walled carbon nanotubes (bMWCNTs) dispersion in polyethylene imine. Then the GCE/bMWCNTs-PEI electrode was treated with AuNPs-B(OH)_2_ and the resulting hybrid surface was used for GOx immobilization. This biosensor exhibited a sensitivity of 28.6 mA M^−1^ cm^−2^ and showed reasonable levels of stability and reproducibility such that the sensitivity was measured to be some 86.1% of the original value after 14 days of storage. The goal of such work was to design a novel hybrid nanomaterial by integrating the inherent advantages of the chosen components, namely gold nanoparticles and carbon nanotubes. Thus, the presence of boronic acid residues allowed the easy immobilization of the enzyme while the bMWCNTs-PEI dispersion provided the best platform for the transduction of the electrochemical response.

Turner’s group have demonstrated the formation of structured layers of gold nanoparticles on two-dimensional molybdenum disulfide (MoS_2_) nanosheets which has gained attention very recently in the field of electrochemical biosensors as an ideal candidate for matrix development [48]. Briefly, a dispersion of MoS_2_ nanosheets in PBS was prepared by ultrasonic treatment and then the dispersion of MoS_2_ was mixed with a commercial 5 nm diameter gold nanoparticles solution. By incubating the mixture at room temperature for 3 h, MoS_2_/AuNPs self-assembled nanosheets were prepared. The resulting MoS_2_/AuNPs were mixed with glucose oxidase enzyme and incubated overnight. The MoS_2_/AuNPs/GOx hybrid structure was assembled onto a gold electrode surface by drop-casting. The biosensor showed a sensitivity of 13.80 µA mM^−1^ cm^−2^. The developed bio-catalytic interface based on two-dimensional MoS_2_ and gold nanoparticles has the potential to be used for the immobilization of other biomolecules [49,50,51,52].

**Figure 5:**
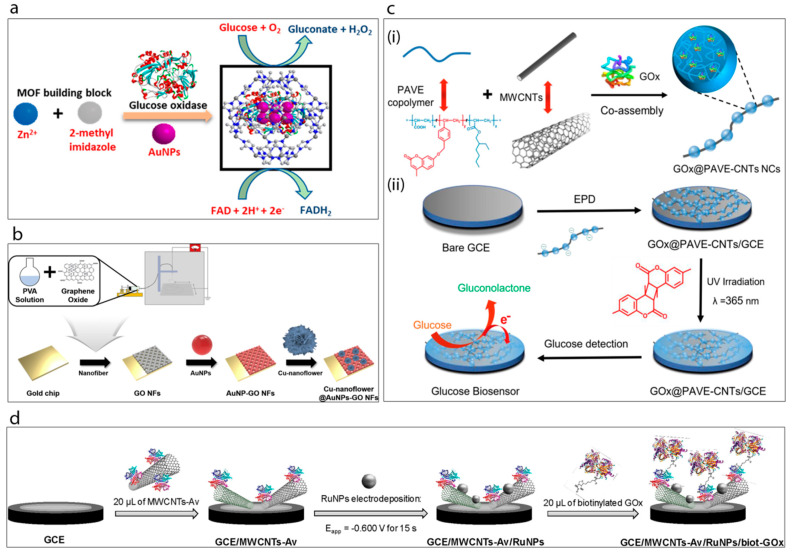
(**a**) Synthesis of GOx@ZIF-8(AuNPs) [53]. (**b**) Schematic display of the fabrication of Cu-nanoflower@AuNPs-GO NFs-based electrochemical glucose nano-biosensor [16]. (**c**) Schematic illustration of the enzymatic glucose biosensor fabrication [12]: (i) preparation of the long conducting enzyme-loading hybrid nanocomposite GOx@PAVE-CNTs via one-step co-assembly; (ii) preparation process of glucose biosensor via direct electrophoretic deposition (EPD) of GOx@PAVE-CNTs onto glassy carbon electrode (GCE) surface and subsequent photo-cross-linking. (**d**) Schematic display of the steps involved in the preparation of the GCE/MWCNTs-Av/RuNPs/biot-GOx biosensor [54].

A new method has been developed for the encapsulation of gold nanoparticles and glucose oxidase together into the cavity of a zeolitic imidazole framework (ZIF-8) [53], Figure 5a. This study shows that the ZIF-8 is stable and provides a very large surface area with a unique cavity which can accommodate both AuNPs and GOx. Furthermore, the AuNPs were found to promote the electron transfer efficiency of the system due to their high conductivity and for this reason the authors claim that the incorporation of AuNPs improved the sensitivity of the system by up to 10-fold.

Baek et al. [16] have very recently reported a study of gold nanoparticles, graphene oxide and copper nanostructures incorporated into a glucose biosensor, Figure 5b. The biosensor matrix was established on a gold chip. A solution of poly(vinyl alcohol) (PVA) was prepared and mixed with graphene oxide (GO) under constant stirring conditions. The prepared mixture was then spin-coated on the surface of the gold chip to obtain GO/PVA nanofiber. This was followed by the attachment of cysteamine-modified gold nanoparticles onto the surface of the fibers. Meanwhile, Cu-nanoflowers were synthesized using a mixture of glucose oxidase, horse radish peroxidase and CuSO_4_ in PBS which was incubated at room temperature for 72 h and then washed with PBS several times. The mechanism of nanoflower formation was explained in terms of both nucleation and growth processes. Basically, the formation of the Cu-protein complex acts as a seed for the nanoflower and these nuclei grow over the reaction time and form the petals of the flower. The as-prepared nanoflowers were dropped onto the AuNPs-GO/PVA electrode surface. This biosensor exhibited the best activity at pH 5 with very good associated analytical performance.

#### 2.1.2. Carbon Nanotubes (CNTs) and Their Hybrid-Based Glucose Biosensors

Since their early discovery in 1991 [55], carbon nanotubes have attracted enormous interest from researchers studying many different fields [56,57,58,59,60]. A considerable amount of work has been performed in which CNTs have been incorporated into electrochemical sensors and biosensors; thus, in this review only some of the most promising examples of CNT-glucose biosensors are described.

Yu et al. [13] have demonstrated the preparation of poly (diallyldimethylammonium chloride) (PDDA)-capped gold nanoparticles (AuNPs) which were then combined with functionalized graphene (G)/multi-walled carbon nanotubes (MWCNTs) to form a nanocomposite which was then used as an immobilization matrix for GOx enzyme. This biosensor exhibited a sensitivity of 29.72 mA M^−1^ cm^−2^ and showed a very satisfactory associated analytical performance towards glucose since the graphene-nanotube and gold nanoparticle composite hierarchical structure provided a conductive network for efficient electron transfer as well as providing more binding sites for the enzyme.

Vilian and Chen [61] developed a glucose oxide biosensor based on multiwalled carbon nanotubes which was modified with the biopolymer L-arginine. MWCNTs were treated with strong acids to produce surface carboxyl groups and then the functionalized MWCNTs (f-MWCNTs) were drop-casted onto a clean glassy carbon electrode. Following this a poly(L-arginine) film was formed on the f-MWCNTs-modified GCE via electro-polymerization. The as-prepared P-L-Arg/f-MWCNT/GCE electrode was then treated with GOx solution in order to immobilize the enzyme. The sensitivity of this biosensor was found to be 48.86 μA mM^−1^ cm^−2^ with a good storage stability of 25 days.

Xu et al. [12] developed a highly interesting nanocomposite, a so-called “necklace-like” material, by the facile one-step co-assembly of the GOx, a copolymer and MWCNTs, Figure 5c. For this purpose, the copolymer poly(acrylic acid-r-(7-(4-vinylbenzyloxy)-4-methyl coumarin)-r-ethylhexyl acrylate) (PAVE) which contains photo-cross-linkable coumarin segments and carboxylic groups was co-assembled with MWCNTs, while simultaneously encapsulating the GOx. This preparation process generated enzyme-loaded polymeric nano beads attached along the length of the MWCNTs. Then, the resulting GOx@PAVE-CNTs bio-nanocomposites were electrodeposited onto a glassy carbon electrode surface followed by a photo-cross-linking process induced by UV irradiation. In this way a robust, complex, biosensing film having a porous network was achieved. The resulting biosensor exhibited highly satisfactory analytical performance towards glucose in terms of linear range (0.001–1.0 and 1.0–5.0 mM), stability (35 days) and reproducibility (RSD 3.27%, *n* = 5).

Recently, Rivas’s group [54] have demonstrated an original supramolecular architecture based on a rationally-designed nanohybrid combination of MWCNTs and ruthenium nanoparticles (Figure 5d). For this construction they took advantage of avidin which is a biotin-binding protein. Briefly, MWCNTs were functionalized by avidin creating MWCNTs-Av which then served as a platform for the immobilization of a biotinylated glucose oxidase enzyme. The clean glassy carbon electrode was drop-cast with as-prepared MWCNT-Av complex and this was followed by electrodeposition of Ru nanoparticles. This GCE/MWCNTs-Av/RuNPs electrode with avidin terminals was found to be highly suitable for the attachment of the biotinylated enzyme complex which was achieved by incubating the electrode with biot-GOx solution. It was shown that the resulting developed surface was highly sensitive towards hydrogen peroxide. Then it was proven that the final GCE/MWCNTs/Av/RuNPs/biot-GOx biosensor) showed a sensitivity of 2.60 μA mM^−1^ cm^−2^ towards glucose. The authors claim that their platform acts as a pseudo-bienzymatic glucose biosensor where glucose was first oxidized by GOx and the resulting hydrogen peroxide was then reduced by the MWCNTs-Av/RuNPs complex.

#### 2.1.3. Carbon/Graphene Quantum Dots (CQDs, GQDs)-Based Glucose Biosensors

Carbon or graphene quantum dots are a relatively new class of carbon-based nanomaterials. Since their discovery in 2004, they have attracted considerable attention from researchers due primarily to their highly interesting photoluminescence (PL) behavior. At the time of writing the use of such tiny nanostructures in the field of electrochemical biosensors is limited. Apart from their PL properties, carbon/graphene quantum dots have several other interesting properties associated with their very small size, their ability to be readily functionalized, their relatively cheap, easy methods of preparation and their ability to disperse readily in water [19,20,62,63]. These benefits combined with the fact that they are essentially non-toxic make them highly promising candidates as components of a biosensing matrix.

For instance, it has been reported that the graphene quantum dots are highly suitable for use as substrates for the glucose oxidase immobilization process [18]. In this report, the graphene quantum dots were prepared by a hydrothermal method from graphite powder. The as-prepared graphene quantum dot solution was drop-casted onto a ceramic carbon electrode surface and dried at room temperature. The resulting GQDs electrode was then activated at a potential of 1.7 V (vs. SCE) and followed by glucose oxidase immobilization. The sensitivity of this relatively simple biosensor based on GQDs was reported to be 0.085 μA μM^−1^ cm^−2^, while it showed reasonable stability over a two-week period.

**Figure 6:**
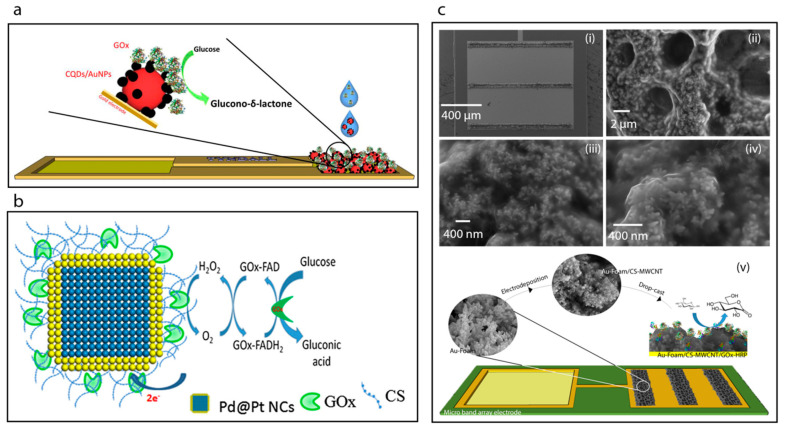
(**a**) Schematic image the construction of the CQDs/AuNPs-GOx biosensor [20]. (**b**) Schematic displaying the surface modification of Pd@Pt NCs using chitosan solution (CS) biopolymer and the covalent immobilization of GOx to the CS by reacting with GA to cross-link the amino group of CS and the FAD site of GOx [30]. (**c**) SEM images showing the Au-foam band array after CS-multi-walled carbon nanotubes (MWCNT) electrodeposition (i), the Au-foam/CS-MWCNT pores (ii) and SEM images of the CS-MWCNT nanocomposite film electrodeposited nanostructures with higher magnification (iii,iv), and construction of the Au-foam/CS-MWCNT/HRP-GOx microbiosensor (v) [14].

Very recently, Buk et al. [19,20] reported follow-up studies for electrochemical glucose biosensing by using gold nanoparticle/carbon quantum dots (AuNP/CQDs) nanohybrid-modified microfabricated gold electrodes. Briefly, they prepared a hybrid combination of AuNP/CQDs based on carbodiimide chemistry attachment and in so doing achieved the covalent attachment of the CQDs on the surface of the AuNPs. Then the nanohybrid structures developed were used as an immobilization matrix for glucose oxidase enzyme by using a microfabricated planar gold electrode (Figure 6a) [20] and a microfabricated micro disk array electrode [19]. The sensitivities the planar and micro biosensor was reported to be 47.24 µA mM^−1^ cm^−2^ and 626.06 µA mM^−1^ cm^−2^, respectively. Both sensors exhibited excellent analytical performance towards glucose.

#### 2.1.4. Hydrogel-Chitosan-Based Glucose Biosensors

Chitin and chitosan are natural polyaminosaccharides. Chitosan is obtained by controlled N-deacetylation of chitin. Chitosan is insoluble in water; however, the amino groups render it soluble in acidic solution below pH 6.5. Most importantly it has an ability to form hydrogels and it is possible to produce thin hydrogel films of chitosan on solid electrode surfaces via a controlled electrodeposition process which makes it particular applicable to the fabrication of miniaturized biosensors. Moreover, the abundant amine groups of the chemical structure make it a useful candidate for applications where various surface chemical methods are required in order to achieve immobilization of a particular biomolecule. As a result of these benefits together with its biocompatibility and lack of toxicity, chitosan has been widely used not only in the field of electrochemical biosensors [64] but also in controlled release systems [65,66], drug delivery applications [67,68,69], wound dressings [70] and tissue engineering [71,72,73].

Here, we focus on the fabrication and characterization of glucose oxidase-immobilized chitosan matrixes. For example, Krishnan et al. [30] utilized chitosan as an immobilization matrix for glucose oxidase enzyme, Figure 6b. Briefly, Pd nanocubes were synthesized with an average edge length of 13 nm and then a shell of Pt was deposited on their surfaces. The as-prepared nanocubes of Pd-Pt were then incubated with a chitosan solution to achieve the chitosan coating. The covalent immobilization of glucose oxidase on the surface of chitosan coated nanocubes was accomplished by reacting with gluteraldehyde followed by addition of the glucose oxidase enzyme. The GOx-immobilized chitosan-coated nanocubes were then deposited on a glassy carbon electrode surface. The sensitivity of the resulting biosensor was determined to be 6.82 μA mM^−1^ cm^−2^.

In another study chitosan was used as a protection barrier for the immobilized enzyme to ensure the stability and biocompatibility of the biosensor [74]. For this purpose, a gold electrode was modified with cysteamine to obtain an amine-functionalized surface and then a solution of glucose oxidase enzyme was drop-casted onto the electrode which was then allowed to dry. This process was repeated several times. Then, chitosan solution was coated onto the glucose oxidase layer. It was found that the response of the resulting biosensor response remained almost constant over a 30-day period, with the RSD range from 1.3% to 7.2%. The stability of the biosensor was attributed to the chitosan protection layer.

Anusha et al. [75] developed a glucose oxidase biosensor based on the use of chitosan nanoparticles taken from a squid. Briefly, the squid was dried at room temperature after removal of debris and then powdered to extract the chitin by deproteinization and demineralization processes. The chitin obtained was then treated to prepare the chitosan by a deacetylation process and the resulting chitosan was used to prepare chitosan nanoparticles. To construct the biosensor, electrodes were first covered with gold nanostructures to increase the surface area, and then these electrodes were immersed into a solution of chitosan nanoparticles. The resulting surfaces were then used to immobilize glucose oxidase enzyme. The resulting biosensor exhibited a high sensitivity of 156.27 μA mM^−1^ cm^−2^ with good associated analytical performance. The results were attributed to the presence of a chitosan nanoparticles matrix over the gold nanostructures which created a friendly environment for enzymes and enhanced the catalytic activity towards glucose.

Another approach utilizing chitosan as a matrix component for glucose biosensor development was reported based on a pyrrole-branched-chitosan [76]. This polymer composite structure was prepared via carbodiimide chemistry. The carboxylated pyrrole was attached to the amine groups of chitosan via amide binding. A chitosan-pyrrole-gold-GOx nano-biocomposite was then prepared by mixing and incubating on the electrode surface. It was found that the chitosan-pyrrole (hydrogel-conductive polymer) composite was a useful host for the immobilization of biomolecules as well as acting as an in situ reducing agent for the formation of gold nanoparticles on the electrode surface.

The electrodeposition of chitosan films is one of the common methods now used in biosensor development [77,78,79]. For example, very recently, Juska et al. [14] have reported a biosensor towards glucose based on a two-step electrodeposition process, Figure 6c. Firstly, these workers achieved the deposition of gold foam on to a microfabricated gold electrode array in acidic environment at high negative applied voltage. Then, an organic functional layer was electrodeposited on to the gold foam surface which consisted of chitosan and multiwalled carbon nanotubes. The biosensor was constructed on a microfabricated band gold electrode array and exhibited excellent analytical performance with a sensitivity of 261.8 μA mM^−1^ cm^−2^ and a reproducibility standard deviation (RSD) of 3.30%.

Che et al. [80] reported a simple one-step deposition of chitosan in the presence of MWCNTs, hollow PtCo nanochains and the dye, Prussian blue. The electrodeposited hybrid film was deployed as a glucose oxidase immobilization matrix and then coated with nafion film. Encapsulation of MWCNTs and Prussian blue in the chitosan gel layer was found to improve the electron transfer ability of the three-dimensional matrix in comparison to the Prussian blue-encapsulated chitosan matrix alone. The authors attributed these enhanced electrical characteristics to the interactions between the MWCNTs, the hollow PtCo nanochains and the Prussian blue molecules.

Table 1 summarizes the very recent literature examples of other essential enzymatic glucose biosensors based on different nanostructures and polymers. Enzymatic biosensors towards glucose have been advancing with the integration of smart nanomaterials. However, their excellent analytical performance of developed enzymatic glucose biosensors has been depended to certain pH ranges, temperatures and also humidity [81].

### 2.2. Non-Enzymatic Detection of Glucose; Direct Glucose Electro Oxidation

Glucose oxidase-based biosensors have been studied extensively (as discussed above); however, the possible decrease in catalytic activity of the enzyme arising from the immobilization process is still a great challenge for researchers in terms of the performance of the sensor as well as the long-term stability of the desired biosensor [94,95]. Direct electro-oxidation of glucose in the absence of the enzyme may provide a solution to some of the problems of enzymatic systems. However, the selectivity of the catalysts developed towards glucose should be investigated in detail if the biosensor is to be used in a highly complex matrix. It is noteworthy that at the time of writing most enzyme-free biosensors for glucose detection exhibit sensitivities at the highest end of the scale in comparison to the enzyme-based glucose sensors [23,96,97,98].

**Figure 7:**
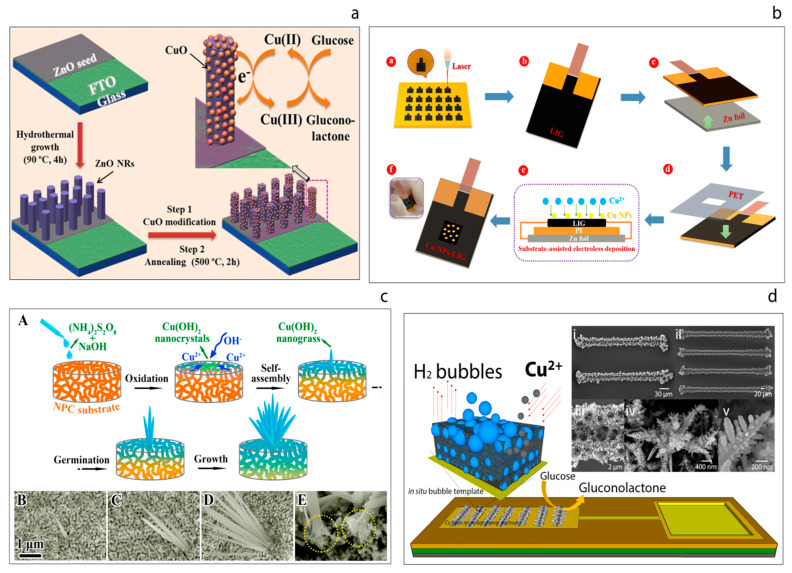
(**a**) Schematic illustration of non-enzymatic glucose sensor electrode fabrication and its application in glucose detection [99]. (**b**) Schematic illustration of fabrication process of the flexible Cu NPs-LIG sensor [100]. (**c**) Schematic illustration of the growth process of the Cu(OH)_2_ nanograss structure on a NPC substrate (A), the corresponding surface SEM images at different growth stages (B–D), magnified cross-section of the fabricated nanohybrid (E) [101]. (**d**) Hydrogen bubble template-based electrodeposition process of the copper foam and the SEM images of the resulting CuFoam electrodeposits [23].

For example, a biosensor based on a simple nanohybrid composition of ZnO nanorods and CuO nanoparticles was reported in 2017 which showed excellent analytical performance [99], yielding a sensitivity of 2961.7 μA mM^−1^ cm^−2^ (Figure 7a). In this study, vertically-aligned ZnO nanorods were grown on fluorine doped tin oxide (FTO) electrodes. The hydrothermally-grown ZnO nanorods on the electrode surface were then modified with CuO nanostructures through a dip-coating and annealing process. The resulting hybrid CuO-ZnO material was coated with nafion to reduce possible fouling and to help limit possible interference effects. The resulting hybrid sensor exhibited a sensitivity of 2961.7 μA mM^−1^ cm^−2^ with excellent reproducibility, repeatability, stability and selectivity. Furthermore, the sensor was also used to determine the glucose concentration in real human serum samples. The high performance of the sensor was attributed to the efficient electrocatalyst behavior of the CuO-ZnO hybrid material for glucose oxidation.

Recently, a flexible non-enzymatic glucose biosensor based on a laser-induced graphene electrode modified with copper nanoparticles has been reported [100]. The flexible graphene electrodes were prepared by laser irradiation of the surface of a sample of polyimide as shown in Figure 7b. Since high-intensity laser radiation is applied, the polyimide is essentially depolymerized, which leads to subsequent carbonization and eventual graphitization. The resulting three-dimensional porous laser-induced graphene (LIG) electrodes obtained were modified with Cu nanoparticles (Cu NPs). The as-prepared Cu NPs-LIG sensor demonstrated a glucose sensitivity of 495 μA mM^−1^ cm^−2^. The authors refer to the use of such a ‘simple method’ for the fabrication of a sensor device and suggest that their approach could be attractive in terms of the fabrication of next-generation flexible diagnostic devices, although the need for high intensity laser irradiation rather calls this presumption into question.

Another recent study based on the use of a novel uniform composite of an Au nanostructured honeycomb coated with a layer of Co_3_O_4_ nanoneedles appeared to be a highly promising catalyst for glucose oxidation [102]. These authors achieved the deposition of the highly porous honeycomb-like gold nanostructures onto the electrode surface via electrodeposition in acidic environment. The synthesis of Co_3_O_4_ nanoneedles on the surface of the honeycomb-like gold was then achieved by a hydrothermal synthesis process in an autoclave and subsequent annealing at 450 °C. The resulting hybrid electrode was studied to determine its performance as a glucose sensor and exhibited a sensitivity of 2013.6 μA mM^−1^ cm^−2^ with a good selectivity towards glucose in alkaline media for both blood and synthetic saliva.

Another interesting non-enzymatic approach has been reported which involves the synthesis of Cu-Cu_2_O nanoparticles on the surface of TiO_2_ nanotubes which act as transducers for the direct electrocatalytic oxidation of glucose [103]. The TiO_2_ nanotubes were prepared by the two-step anodization of Ti foils and then the preformed Cu and Cu_2_O particles were coated onto the TiO_2_ nanotubes by an electrodeposition technique. The resulting hybrid electrode was then used for the non-enzymatic electro-oxidation of glucose. The sensitivity of sensor was calculated to be 4895 μA mM^−1^ cm^−2^. Such a high sensitivity was attributed to the synergistic effect of the small Cu-Cu_2_O grain size and the very large surface area of the TiO_2_ nanotube arrays as well as to fast electron transfer. Moreover, by evaluating the results obtained from studies of the various deposition parameters used, the authors claim that the Cu_2_O helps to provide a broad linear range while the incorporation of the Cu nanoparticles helps to improve the response current and sensitivity.

Li et al. [101] have reported a novel hybrid non-enzymatic glucose sensor consisting of a freestanding Cu(OH)_2_ nanograss array on the surface of a nanoporous copper (NPC) substrate, Figure 7c. These authors first prepared the nanoporous copper substrate from a CuZrAl glassy precursor via a chemical de-alloying process. Then, the Cu(OH)_2_ nanograss was synthesized on the NPC substrate through an oxidative alkaline method, wherein the morphology of the nanograss was tailored by varying the etching time. The substrate was placed into a solution of (N_2_H_4_)_2_S_2_O_8_ and NaOH until the surface color turned light blue. This process was explained in terms of four stages; oxidation, self-assembly, germination and growth. The resulting uniform hybrids also grew homogenously. It was found that the nanograss clusters exhibited high performance towards the oxidation of glucose, with a sensitivity of 2.09 mA mM^−1^ cm^−2^ being recorded.

Juska et al. [23] have demonstrated the miniaturization of the copper foam (CuFoam) nano dendrites and their use for glucose electro-oxidation, Figure 7d. These authors firstly fabricated two different band gold electrodes arrays at micro scale and this was followed by the electrodeposition of CuFoam by applying negative high voltages in acidic solution in the presence of Cu^2+^ ions. The study explains the surface composition changes of copper oxide species before and after glucose oxidation process. Furthermore, the sensor developed exhibited superior analytical performance with a sensitivity of an outstanding sensitivity of 10.630 μA mM^−1^ cm^−2^ toward glucose with a wide linear range up to 22.55 mM.

Table 2 provides literature examples on non-enzymatic glucose sensing platforms.

## 3. Wearable Non-Invasive Electrochemical Glucose Sensors

The majority of detection technologies for determination of glucose concentration rely on blood or serum analysis. It is also possible to detect glucose from the other bodily fluids such as sweat, saliva and tears. For this purpose, in particular, noninvasive glucose sensing platforms are in great interest since they may be the ideal candidates for diabetes management. Such sensors do not contact with the blood thus they are not exposed to the immune system, as it happens in implantable glucose sensors. However, the major problems related to wearable systems arise from physiological nature of external bodily fluids of tears, saliva and sweat [113,114] and due to the challenge of reproducible sample collection.

**Figure 8:**
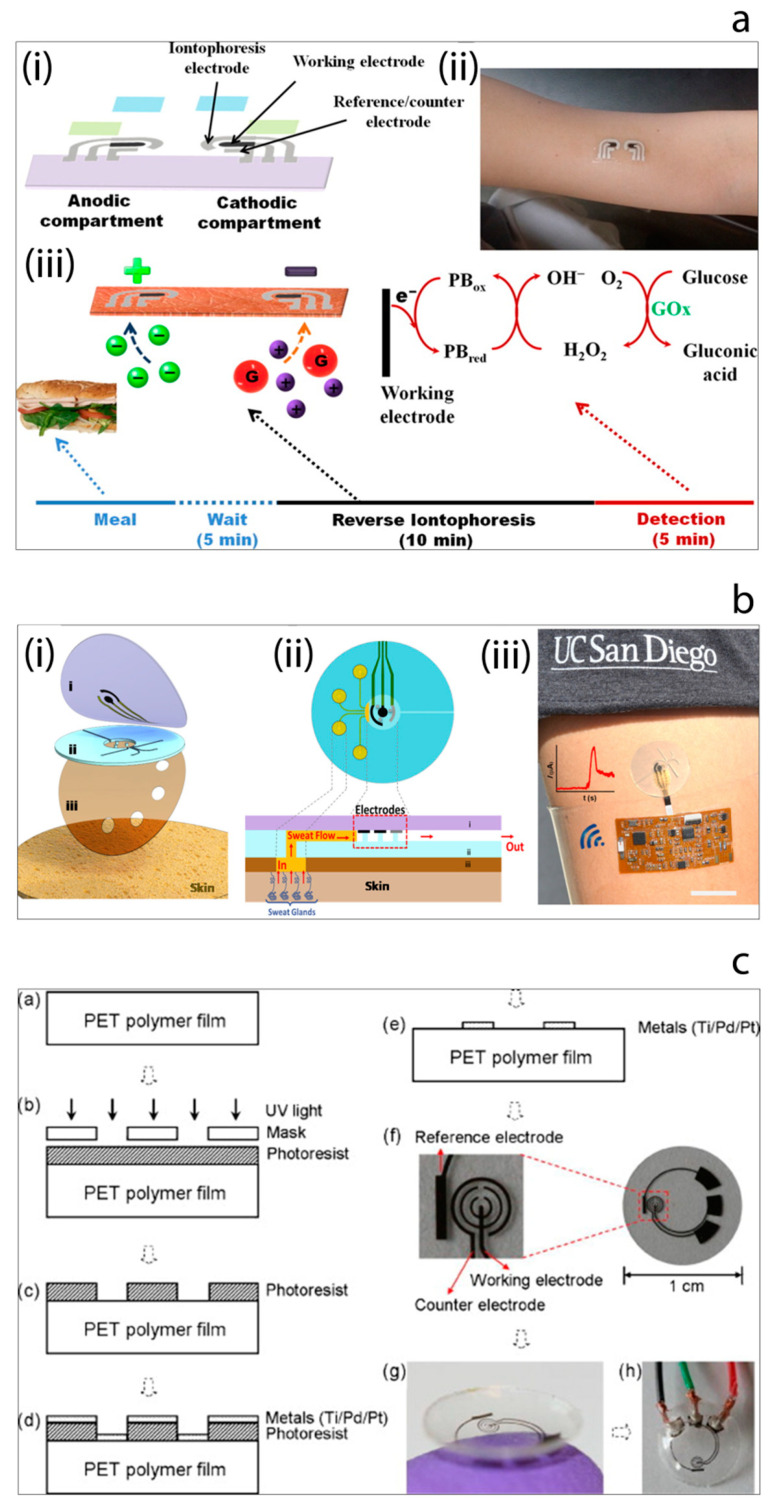
(**a**) Tattoo-based noninvasive glucose sensing platform: (i) schematic image of the printable iontophoretic-sensing system, (ii) photograph of the tattoo-based glucose sensor device applied to a human object, (iii) schematic image of the time frame of a typical on-body study [115]. (**b**) Microfluidic device design and operation: (i) schematic image of the layered microfluidic device configuration, (ii) schematic representation of microfluidic device sweat collection and operation on skin, (iii) photograph of the device integrated with wireless conformal electronics on skin [117]. (**c**) The contact lens sensor fabrication process and resulting contact lens with embedded sensor [118].

In order to overcome such drawbacks, a tremendous amount of efforts have been made by researchers in the field. For instance, Wang et al. [115] have reported the development of a proof-of-concept non-invasive glucose sensing platform based on all-printed temporary tattoo, Figure 8a. The system used a low current density to extract the skin interstitial fluid (ISF) to decrease the skin irritation and selective sensing of glucose by using glucose oxidase-modified Prussian Blue transducer was achieved at a low applied potential. The authors also examined the on-body performance of the tattoo-based iontophoretic-biosensing platform to compare the changes in the glucose level pre- and post-meal. Chen et al. [116] have designed and fabricated an ultrathin flexible skin-like biosensor system based on a biocompatible paper battery with gold electrode for non-invasive, in situ and accurate monitoring of intravascular blood glucose. They used hyaluronic acid to increase the ISF osmotic pressure to promote the intravascular blood glucose refiltration. The resulting biosensors exhibited a sensitivity of 130.4 µA/mM and were conducted in vivo human clinical trials. The obtained results showed a high correlation with clinically measured glucose concentrations. In 2017, Wang et al. [117] have also reported another study which described a flexible epidermal microfluidic detection platform fabricated by integration of lithography and screen-printing technologies in order to achieve effective sweat sampling for continuous glucose and also lactate monitoring, Figure 8b. Developed wearable micro device successfully enabled efficient sweat pumping process to the electrochemical detection chamber consisted of enzyme-modified electrode transducers. Furthermore, device was examined with healthy human subjects to illustrate its sensing ability. Yao et al. [118] demonstrated the fabrication and the use of a contact lens with embedded sensor for glucose measurement from tear, Figure 8c. For the construction of the contact lens, a PET polymer is used as a substrate and spin-coated with a resist. This is followed metal deposition and lift-off in acetone. Fabricated electrodes are cut to small pieces with 1 cm diameter and heat molded to the shape of the contact lens. To achieve the immobilization of glucose oxidase enzyme, a solution of enzyme is drop-casted on the electrode surface, and then the surface is suspended vertically above a titanium isopropoxide solution in a sealed dish to create glucose oxidase/titania sol-gel membrane. After forming sol-gel membrane, surface is covered with nafion. Developed non-invasive glucose monitoring system is studied with amperometry. Such simple micro-sized glucose sensor fabricated on a polymer contact lens showed a good sensitivity of 240 μA cm^−2^ mM^−1^, however many characteristics remain to be improved such as stability, full biocompatibility for wearable contact lens, integration with a read-out-communication circuit.

Non-invasive wearable electrochemical glucose sensor development has been extensively studied to improve the analytical performance of the measured glucose signal and to achieve the validation of the sensors with blood glucose concentrations. Developments related to integrated systems with body-compatible flexible substrates and also improved collection protocols of target bodily fluids have contributed to the success of the wearable devices for glucose monitoring. However, there are still challenges which require extensive studies to overcome for the construction of the excellent wearable devices for glucose sensing such as inconsistent biofluid extraction, surface contamination and the effect of the physiological conditions on the accuracy of the obtained signals. Noninvasive wearable glucose sensors have also gained attention of the companies which develop technologies for this purpose.

## 4. Point-of-Care Diagnostic Devices Based on Personal Glucose Meters

The very successful launch of the first commercial glucose meter of YSI opened up a new era for the point-of-care diagnostic tools. Since then many companies have released their own version of personal glucose meters including an electronic device and suitable sensing probe. The success of the home blood glucose meters improved the control of diabetes by allowing the medical professionals to diagnose or treat the patients without a need to resort to analytical laboratories [119]. It is worth mentioning that accurate, portable devices could prevent millions of deaths each year since they could also improve the management of transmissible diseases and life-threatening conditions [119,120]. The very recent example of the Covid-19 pandemic has also proven the importance and the urgent need for accurate detection technologies to be used on-site to monitor the health of a population. Moreover, such devices provide a quantitative analysis of the target, thus providing information about the status or stage of the disease. However, the development and commercialization of other medical point-of-care devices has not paralleled the revolutionary success of home blood glucose due to the high cost and the necessary time to bring one to the market.

**Figure 9:**
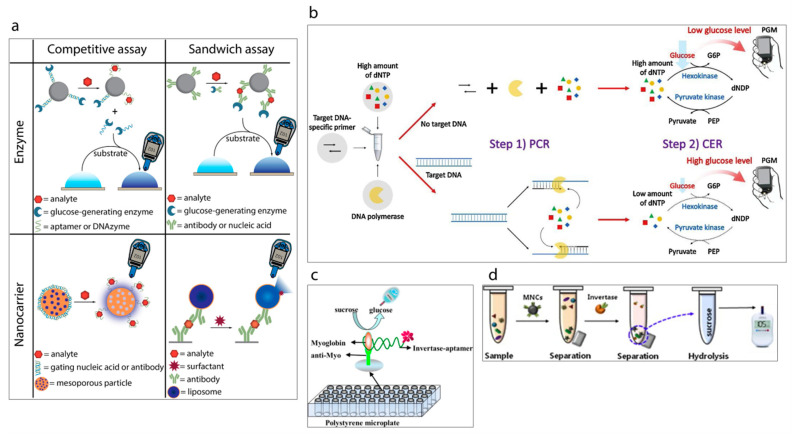
(**a**) Classification of the strategies to detect analytes other than glucose using a personal glucose meter [119]. (**b**) Schematic illustration of PGM-based label-free strategy for target DNA detection [123]. (**c**) Schematic illustration of myoglobin detection using aptamer and personal glucose meter [133]. (**d**) A schematic illustration of the experimental procedure using a personal glucose meter for the detection of *Salmonella* bacteria [129].

Recently, a new trend based on repurposed-personal glucose meters for the detection of specific targets other than glucose has been gaining attention [121] (a summary of detection strategies is shown in Figure 9a [119]). A pioneering example of this initiative has been reported by Lu and Xiang [122] in 2011 in which a broad range of non-glucose targets were successfully detected by using a commercial glucose meter. The methodology developed methodology links the concentration of targets with the concentration of measured glucose via glucose meter. This is achieved with the approach of target-induced release of invertase enzyme from a functional-DNA-invertase bioconjugate. Then, the released enzyme converts sucrose into glucose which can be detected with a glucose meter. This first report of re-purposed glucose meter applications was followed by the other researchers, who studied this approach using different analytes including DNA [123,124,125], protein [126,127], enzyme [128], bacteria [129,130], and heavy metals [131,132], e.g., very recently Kim et al. [123] have reported the development of a label-free target DNA detection protocol based on a glucose meter which consists of a cascade reaction scheme of two different enzymes, namely hexokinase and pyruvate kinase, Figure 9b. The system developed is able to link the concentration of deoxynucleoside triphosphate (dNTP) with the concentration of glucose which is measured by glucose meter. Wang et al. [133] have developed a sensing platform towards myoglobin which is a cardiac biomarker by using an aptamer as a recognition element, Figure 9c. Briefly, target myoglobin first was captured by specific myoglobin antibodies which were immobilized on the surface of a polystyrene microplate. This was followed by the conjugation of the myoglobin specific aptamer with invertase enzyme. Thus, the aptamer could capture the attached myoglobin and subsequently the obtained sandwich assay could hydrolyze sucrose into glucose via the invertase enzyme. The resulting glucose concentration was detected rapidly by the glucose meter. Such sensing protocols can also be applied to detect pathogens. The study published by Joo et al. [129], demonstrates the transformation of personal glucose meter into a sensitive method for detection of pathogenic bacteria *Salmonella* from a complex matrix of milk as shown in Figure 9d. For this purpose, firstly superparamagnetic Fe_3_O_4_ nanoclusters were synthesized and modified with anti-*Salmonella* antibodies to separate the target *Salmonella* from the sample. Then, the resulting magnetic nanocluster-*Salmonella* complexes were conjugated to polyclonal antibody functionalized invertase enzyme to achieve the hydrolysis of sucrose to glucose. Thus, the glucose concentration could be determined by the personal glucose meter which was linked to the concentration of the captured Salmonella by the magnetic nanoparticles.

The detection of non-glucose targets via a point-of-care glucose meter is an area which is rapidly gaining in popularity which aims to develop sensitive detection platforms which could be used without any prior professional knowledge. One of the challenges in this research field is the simplification of the sample preparation step since the developed methods are not as simple as glucose detection from whole blood. Engineering an automated system may improve the sample preparation step. The other drawback of such detection technologies is the variability of the commercial glucose meters. The signal obtained from one commercial glucose meter may differ from another commercial device. Moreover, interference can be the other parameter which requires a further investigation to compare different personal glucose meters. These are some of the significant challenges of the personal glucose meter based non-glucose targeted detection systems. However, the developments of sensing protocols in repurposing the personal glucose meters have proven that such approaches can lead the revolutionary low-cost point-of-care diagnostics for diseases.

## 5. Conclusions

In this review, we have summarized the commercialization history of glucose biosensors and have described recent trends in the related research field of enzymatic, non-enzymatic, wearable non-invasive glucose biosensors as well as repurposed personal glucose meters towards non-glucose targets. Most research has proven the fact that electrochemical biosensors based on advanced nanostructures and miniaturized devices offer important sensing and diagnosis platforms demonstrating high sensitivity and selectivity towards various target analytes. Since the very first discovery of biosensors many breakthrough innovations have been demonstrated in order to improve the characteristics and performance of the desired sensing devices. First, second and third generation glucose biosensors, the use of nanomaterials or integrated polymers matrixes have been described in detail for a number of specific applications. Enhanced sensitivities have been achieved for glucose biosensors by applying the use of emerging nanostructures, hybrid materials and micro-or nano technologies. These include carbon-based nanomaterials such as graphene or graphene derivatives, carbon quantum dots, graphene quantum dots, carbon nanotubes, gold nanostructures, biocompatible hydrogel chitosan and their nano-bio-composites.

Recent advances in nanotechnology research have been accelerating the improvements in the development of enzyme free glucose sensors with excellent analytical performances. In particular metal or metal oxide nanostructures offer enhanced electroactive surface area and superior sensitivities as glucose sensors.

Both enzymatic and non-enzymatic sensing platforms still have challenges to be overcome in terms of limitations in regards to their biocompatibility, lifetime, and selectivity. Since many glucose oxidase-based electrochemical biosensors have been used as idealized model systems for the fabrication of diverse sensing platforms, they have opened up the possibility of their use in a new generation of (integrated) electrochemical biosensors for the detection of several other analytes. For example, a combination of an electrochemical glucose biosensor with advanced DNA technologies on a small microfluidic device has been developed at low cost in order to detect miRNAs [134].

Conventional glucose meters require invasive methods for sample collection since such systems work with human blood or serum. The discomfort and pain arising from these invasive techniques have led researchers to focus on non-invasive sensing platforms which can aim to detect glucose from other bodily fluids including tears, sweat or saliva. These achievements in the field of painless, non-invasive detection of glucose coupled with miniaturized systems can be revolutionary in terms of the control and management of diabetes. Furthermore, such achievements could lead to the development of the point-of-care devices for the other possible target analytes.

The personal glucose meter is perhaps the greatest example of a point-of-care device. Commercially available glucose meters have been widely used due to their small size, easy operation, quantitative results and accuracy. These devices detect glucose from whole blood. However, the smart idea of repurposing the personal glucose meters provides the opportunity to create sensing platforms for non-glucose targets as point-of-care devices. Recently, many methods have been developed to use personal glucose meters for bacteria, DNA, disease biomarkers, etc. detection in the presence of the invertase enzyme. For practical applications, various drawbacks associated with personal glucose meters should be studied in detail, including the limited linear range of those devices and the interference effects of the naturally occurring glucose in the biological samples.

Certainly, there are also many other unexplored strategies. With this in mind, there is still a high demand and expectation for robust, accurate, sensitive, selective and cheap sensing devices particularly for the early-stage detection of biomarkers associated with various cancers and diseases such as Alzheimer’s, multiple sclerosis, etc. It is also noteworthy that the development of sensors for the reliable, continuous, real-time monitoring of glucose with high selectivity and speed also currently presents a massive challenge in the area of diabetes control.

## Figures and Tables

**Table 1:** Other nanomaterials, polymer based enzymatic glucose biosensors.

Enzymatic Glucose Sensors	Sensitivity	Linear Range	Detection Limit	Reproducibility	Life Time	Ref.
GR–CNT–ZnO–GOx	5.36 (±0.072) µA mM^−1^ cm^−2^	0.01–6.5 mM	4.5 (±0.08) µM	RSD 3.24% (*n* = 5)	94.6% peak current was retained even after 4 weeks at 4 °C	[82]
ERGOc–MWCNTd/GOx/Nf	7.95 µA mM^−1^ cm^−2^	0.01–6.5 mM	4.7 µM	RSD 205% (*n* = 7)	The biosensor retained its 90% peak current after one month	[83]
Fc/GOD/Au/SLG	-	0.0005–5000 µM	0.0001 µM	RSD 3.8% (*n* = 6)	After two weeks, the current response retained 81% of the initial response	[24]
Au/GNS-PEI-AuNPs/Glu-GOx	93 μA mM^−1^ cm^−2^	1–100 µM	0.32 µM	RSD 6.7% (*n* = 5)	The biosensor was kept in PBS (0.1 M, pH 7.0) at 4 °C while not in use. The current response maintained 88% of its initial value after 10 days.	[84]
PANI-SDS-F127(1:1)/GOx	485.787 μA mM^−1^ cm^−2^	5–50 mM	3.202 μM	-	-	[85]
Pt-CNT-muc 50%	15 mA M^−1^ cm^−2^	0.002–3.2 mM	3 μM	RSD, 2.2% for the set of evaluated samples	300 days	[86]
GOx/PVA-Fe_3_O_4_/Sn	9.36 µA mM^−1^	0.005–30 mM	8 μM	RSD 4.2% (*n* = 5)	The current response of biosensor is maintained about 81% of its initial response after a month.	[87]
Au–Ni coaxial nanorad array/GOx	778.2 μA mM^−1^ cm^−2^	0.0275–27.75 mM	5.5 μM	-	The measured peak current dropped by approximately 13% after 30 days storage at 4 °C	[88]
CS/GOx–PABA–Au_nano_/Au-plated Au	97.7 μA mM^−1^ cm^−2^	0.002–3.7 mM	0.1 μM	RSD 4.2% (*n* = 5)	The current response of the sensor maintains 85% of the initial current response after 1 month.	[89]
GOx/Pt/rGO/P3ABA	22.01 μA mM^−1^ cm^−2^	0.25–6.00 mM	44.3 μM	RSD 2.58% (*n* = 5)	After 7 days, the storage electrode retained the current response of ca. 86% of the initial response	[90]
GOD/CS-rGO/AuNPs/Pt electrode	102.4 μA mM^−1^ cm^−2^	0.01–2.13 mM	1.7 μM	RSD 3.2% (*n* = 5)	After one month, 15% loss of its initial current response was observed	[91]
GOx/PtNP/PANI/PtE	96.1 μA mM^−1^ cm^−2^	0.01–8 mM	0.7 μM	-	-	[92]
GOx/gold/MoS_2_/gold nanofilm on the polymer electrode	-	10–500 nM	10 nM	-	-	[93]

**Table 2:** Other nanostructures based non-enzymatic high-performance glucose sensors.

Non-Enzymatic Glucose Sensors	Sensitivity	Linear Range	Detection Limit	Reproducibility	Life Time	Ref.
SWCNTs/Cu_2_O/ZnO nanorods (NRs)/graphene	466.1 & 203.1 µA mM^−1^ cm^−2^	0–5.556 & 5.556–11.111 mM	-	-	-	[104]
CuO/PANI-NF/FTO	2800 & 1359 µA mM^−1^ cm^−2^	0.00025–0.28 & 0.28–4.6 mM	0.24 µM	RSD 3.6%	after 11 days, the electrode showed ∼90% of its initial signal	[105]
Ni(OH)_2_/CNT fiber microelectrode	12.2 mA cm^−2^ mM^−1^	20 µM–10.5 mM	0.645 μM	-	-	[106]
CuO/NiO/PANI/GCE	-	20–2500 μM	2 μM	RSD 3.8% (*n* = 5)	a loss of only approximately 10.8% in current response after 15 days	[107]
coral-like Cu micro-/nano-structure arrays	3826 μA mM^−1^ cm^−2^	0.20 μM–1.90 mM	0.04 μM	RSD 2.51% (*n* = 6)	14 days; 11.5% loss in current response	[108]
Au@MIP sensor	-	10^−10^ to 10^−8^ mol L^−1^ and 10^−8^ to 10^0^ mol L^−1^	3 × 10^−12^ mol L^−1^	Although the response of the sensor was nearly identical for the electrode originating from same copolymer and Au@MIPNs solution, these copolymers and Au@MIPNs were individually handmade, resulting that there was imperfect reproducibility from one batch to another.	12 days with only a slight decrease in the current	[109]
NiWO_4_-modified GCE	269.6 μA mM^−1 ^cm^−2^	0.004 μM–4.1 mM	0.18 μM	RSD 2.7% % (*n* = 5)	After 50 consecutive CV cycles, it was found that the glucose sensor retained ca. 97.2% of its initial oxidation peak potential value	[110]
S/NPG/Co_3_O_4_ hybrid microelectrode	12.5 mA mM^−1^ cm^−2^	1 μM–10 mM	5 nM	-	The aging tolerance ensures the S/NPG/Co3O4 hybrid microelectrode to retain ~99.5% of its original current response over a storage period of 15 days at room temperature. Even the S/NPG/Co3O4 hybrid microelectrode stored at room temperature for 4 months still maintains the high capability to analyze serum sample at ultralow concentrations	[111]
CuO NWs with Au NPs	1591.44 μA mM^−1 ^cm^−2^	0.001 mM–44.36 mM	0.3 μA	RSD 5% (*n* = 10)	The sensor response retains 95% (measured on day 30) of its original response (measured on day 0).	[112]
